# Improvement of Genomic Predictions in Small Breeds by Construction of Genomic Relationship Matrix Through Variable Selection

**DOI:** 10.3389/fgene.2022.814264

**Published:** 2022-05-18

**Authors:** Enrico Mancin, Lucio Flavio Macedo Mota, Beniamino Tuliozi, Rina Verdiglione, Roberto Mantovani, Cristina Sartori

**Affiliations:** Department of Agronomy, Food, Natural Resources, Animals and Environment, University of Padua, Legnaro, Italy

**Keywords:** genomic selection accuracy, single-step GBLUP, SNP selection methods, machine learning, local breed cattle, Rendena, genomic selection

## Abstract

Genomic selection has been increasingly implemented in the animal breeding industry, and it is becoming a routine method in many livestock breeding contexts. However, its use is still limited in several small-population local breeds, which are, nonetheless, an important source of genetic variability of great economic value. A major roadblock for their genomic selection is accuracy when population size is limited: to improve breeding value accuracy, variable selection models that assume heterogenous variance have been proposed over the last few years. However, while these models might outperform traditional and genomic predictions in terms of accuracy, they also carry a proportional increase of breeding value bias and dispersion. These mutual increases are especially striking when genomic selection is performed with a low number of phenotypes and high shrinkage value—which is precisely the situation that happens with small local breeds. In our study, we tested several alternative methods to improve the accuracy of genomic selection in a small population. First, we investigated the impact of using only a subset of informative markers regarding prediction accuracy, bias, and dispersion. We used different algorithms to select them, such as recursive feature eliminations, penalized regression, and XGBoost. We compared our results with the predictions of pedigree-based BLUP, single-step genomic BLUP, and weighted single-step genomic BLUP in different simulated populations obtained by combining various parameters in terms of number of QTLs and effective population size. We also investigated these approaches on a real data set belonging to the small local Rendena breed. Our results show that the accuracy of GBLUP in small-sized populations increased when performed with SNPs selected *via* variable selection methods both in simulated and real data sets. In addition, the use of variable selection models—especially those using XGBoost—in our real data set did not impact bias and the dispersion of estimated breeding values. We have discussed possible explanations for our results and how our study can help estimate breeding values for future genomic selection in small breeds.

## Introduction

Genomic information has been successfully implemented in animal breeding due to its effectiveness in bringing significant improvements in accuracy (Blasco and Toro, 2014). These improvements in accuracy can lead to an increase in the rate of genetic gains and have reduced the cost of progeny testing by allowing to preselect animals with great genetic merit early (Meuwissen et al., 2001). Combining these advancements with the progressively reduced cost of genotyping makes single-nucleotide polymorphism (SNP) panels a promising tool to select small local breeds (Biscarini et al., 2015).

SNP marker information allows for better modeling of Mendelian sampling than the traditional pedigree-based best linear unbiased prediction (PBLUP) (VanRaden, 2008a), which used only pedigree information. The genomic BLUP (GBLUP) method was developed to replace the pedigree-based relationships for genomic relationships estimated from SNP markers, which captured the genomic similarity between animals but are limited to the use of only genotyped animals (Habier et al., 2013). In addition, Legarra et al. (2009) proposed a naive method, single-step GBLUP (ssGBLUP), in which genotyped and non-genotyped animals are jointly combined under the assumption that the genomic and pedigree relationship matrixes are multivariate and normally distributed. Due to its straightforward computational approach (Misztal et al., 2013) and unbiased breeding values predictions, compared to the GBLUP with its multistep approach (Masuda et al., 2018), the ssGBLUP has become a routine method for genomic evaluations in many livestock breeds and species (Aguilar et al., 2010; Christensen and Lund, 2010).

However, one major challenge in using (ss)GBLUP remains the accuracy of estimation when phenotyped animals are limited in number, such as in local breeds (Meuwissen et al., 2001). For example, Karaman et al. (2016) reported that GBLUP showed lower performance than that of models using only SNPs selected through a Bayesian hierarchical model as Bayes B and Bayes C, but only when phenotyped animals were few. Indeed, when presented with a small number of animals and many SNP markers (n < p), models that select a number of priority SNPs (variable selection models) and models that assume heterogenous variance can lead to improvements in EBV accuracy. These models can accomplish this by reducing the number of variables to estimate and by preventing overfitting linked to high-dimensional data (Gianola 2013). Frouin et al. (2020) went as far as deriving the prediction accuracy of GBLUP as a function of the ratio n/p, while Pocrnic et al. (2019) regarded the accuracy of GBLUP as not only strictly dependent on the number of SNPs but also on the number of independent chromosome segments.

Several studies thus focused on relaxing the assumption of ssGBLUP that all SNPs must show a common variance by applying different weights to the SNPs when the **G** matrix is calculated. Methods such as weighted ssGBLUP (WssGBLUP) (Wang et al., 2014) were widely reported to outperform ssGBLUP’s accuracy of prediction (Gualdrón Duarte et al., 2014; Gualdrón Duarte et al., 2020; Mehrban et al., 2021; Ren et al., 2021), but their use led to a proportional increase of breeding value bias and dispersion (Mancin et al., 2021b; Botelho et al., 2021; Cesarani et al., 2021; Mehrban et al., 2021).

Moreover, it is unclear how models considering heterogenous variances account for selection since only k-fold cross-validation is usually applied (Zhu et al., 2021). In real-life breeding scenarios, time cross-validation should be considered (Liu, 2010) because this validation method mimics the true accumulation of information across time. The estimated breeding values (EBVs) are in fact used to select young bulls, and after 3–5 years, the bulls will receive daughter information; it is thus desirable that EBVs would highly correlate to the final EBVs. However, the few studies that evaluated the impact of WssGBLUP using time cross-validation with small samples of individuals (e.g., Cesarani et al., 2021) found higher bias and overdispersion. These mutual increases are relevant when a low number of phenotypes and high shrinkage values are present, and the reasons behind the loss of these unbiased properties in heterogenous SNP regression or GBLUP are still not entirely clear.

This issue is not trivial as the bias and the slope of the regression (dispersion) need to be considered, especially when proven, and young animals are mixed in the population as young candidates will have unfair EBVs (Legarra and Reverter, 2017).

Thus, the abovementioned issues of lack of accuracy of ssGBLUP when used in contexts with a few animals have not been conclusively resolved. For this reason, in the present study, we intend to explore alternative methods to improve accuracy in small populations within a single-step framework. A possible solution could come from implementing a naïve approach, where instead of giving each SNP a specific weight, we removed the non-informative ones or variable selection models. Thus, we aimed to investigate the impact, in terms of accuracy of predictions, dispersion, and bias, of reducing the dimensionality of the **G** matrix by constructing it using only a subset of informative markers.

In order to accomplish this, we tried different machine learning and variable selection algorithms with the aim to identify the most informative SNPs by indirect prediction. These algorithms were as follows: least absolute shrinkage and selection operator (LASSO), spike-and-slab LASSO (SSLASSO), recursive feature elimination using ridge regression (RfeRR), recursive feature elimination using support vector machine regression (RfeSVM), and extreme gradient boost (XGBoost).

We aimed to test suitable procedures for genomic estimation by considering both the abovementioned variable selection models ssGBLUP and the predictions of BLUP, classical ssGBLUP, and WssGBLUP. To do that, we created different simulated populations and also considered a local population, the Rendena cattle. We then used different cross-validation methods to assess our results.

## Materials and Methods

**FIGURE 1:**
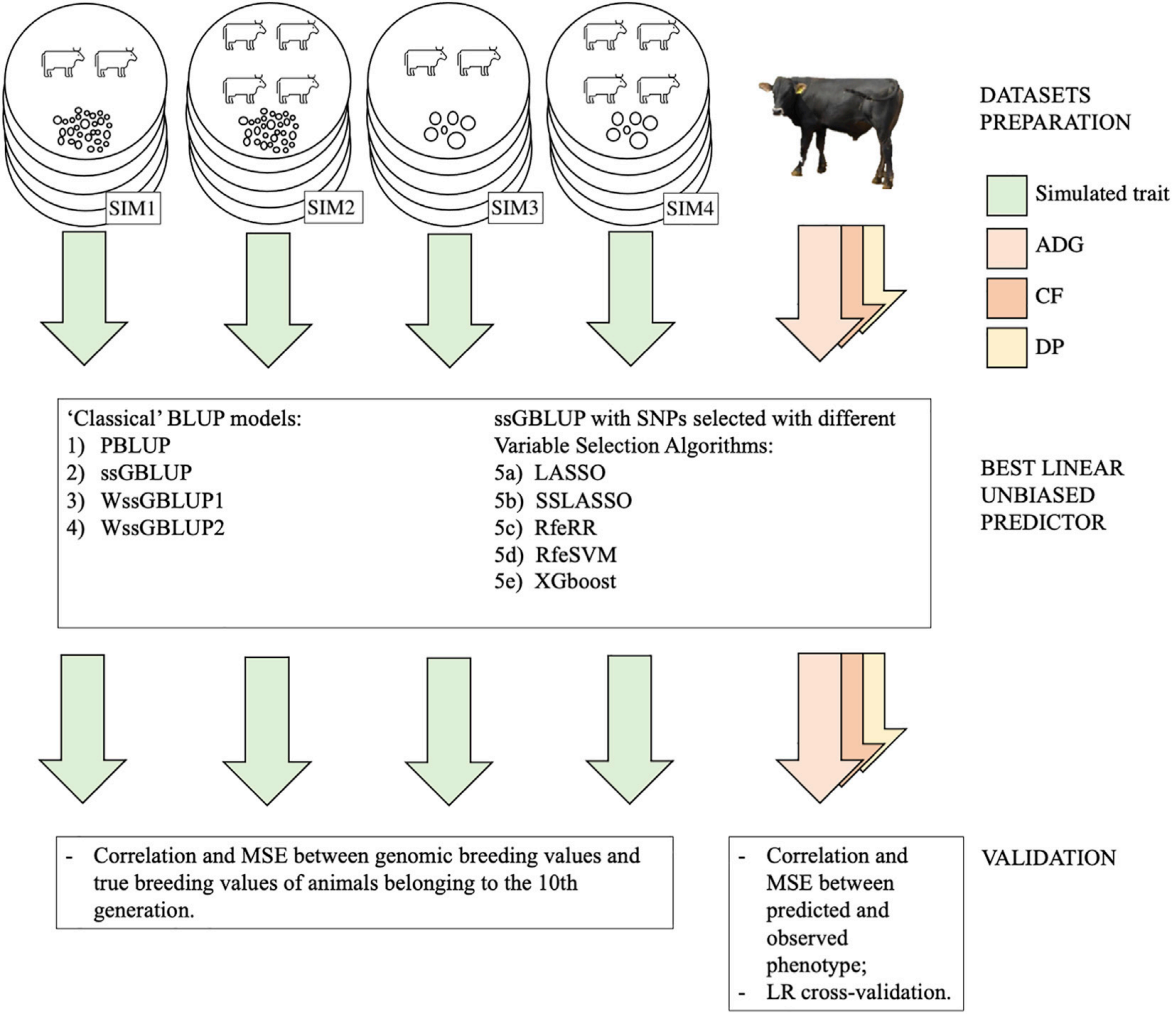
Graphical representation of our methodology for testing model predictions both in simulated and real populations. Each replicate of a simulated population is represented with a circle; SIM1 is polygenic with low Me, SIM2 polygenic with high Me, SIM3 is oligogenic with low Me, and SIM4 is oligogenic with high Me. Each phenotype (both real and simulated) is represented with an arrow.

For a graphical representation of our methodology for testing BLUP models, see Figure 1.

### Data sets

#### Simulated Data sets

Simulations were performed with the QMSim simulation program (Sargolzaei and Schenkel, 2009). A total of four different populations were simulated based on various combinations of quantitative trait locus (QTL) number and effective population size (Ne). Each simulation was replicated five times.

All simulations were generated starting from the historical population using a similar structure to that used by Pocrnic et al. (2019): we created an initial bottleneck contracting the historical population size from 5,000 to 1,000 animals in 1,250 generations and then expanded it to 25,000. In the first generation, 10 bovine autosomes were simulated, placing evenly spaced 80,000 ca. biallelic SNPs with equal allele frequencies and a recurrent mutation rate of 2.5e^−5^ per generation. The number of SNPs per chromosome was set to 8,000, while the QTL number changed according to different simulation strategies. In two of the four simulations, one biallelic and randomly distributed QTL per chromosome was sampled from a gamma distribution with a shape parameter equal to 0.4 (oligogenic scenarios). In the other two simulations, 100 QTLs per chromosome were generated using the same parameter (polygenic scenarios). In all these simulations, 10 discrete generations were created by randomly mating 750 females and a different number of sires according to the simulation strategies. In two scenarios, one oligogenic and one polygenic, we assumed a large Ne, with 100 males per generation used as sires, while in simulations with a low Ne, only 10 males per generation were used as sires. The following four populations were, thus, created by mixing different numbers of QTL and different Ne values, and five replicates for each population were generated:

• SIM1 polygenic population with small Ne
• SIM2 polygenic population with large Ne
• SIM3 oligogenic population with small Ne
• SIM4 oligogenic population with large Ne

The effective population size and number of QTLs in the four different simulated populations are reported in Table 1, and numbers of -genotyped animals are reported in Table 2 (2,250 animals). We simulated a single trait with heritability of 0.3, close to the heritability of the traits in the real data set further described. To do that, we obtained a single phenotype record per animal by adding an overall mean of 1.0 to the sum of the QTL effects together with a residual effect. As in the study by Pocrnic et al. (2019), only phenotypes from generations 8 to 9 were retrieved, and genomic information of animals belonging to generations 8 to 10 was used for further analysis (750 × 3 = 2,250 animals). The structure of simulated populations is reported in Table 2. Before proceeding with genomic prediction, SNPs with a minor allele frequency (MAF <0.01) and with high linkage disequilibrium (LD > 80) were removed using the SNPrune program (Calus and Vandenplas, 2018).

**TABLE 1:** Number of QTLs and effective population size in the four different simulated populations.

	**QTL**	**Ne**
SIM1	1,000	40
SIM2	10	350
SIM3	1,000	40
SIM4	10	350

**TABLE 2:** Population structure of simulated and real data sets.

	**Simulated**		**Real**
	**SIM1–SIM3** ^a^	**SIM2–SIM4** ^a^	
Number of records	1,500	1,500	1,691
Number of animals in the pedigree	3,413	3,794	6,926
Number of genotyped animals	2,250	2,250	1739
Number of genotyped animals with records	1,500	1,500	687
Inbreeding from pedigree	0.0126	0.0009	0.0316

a Since population structure is the same for SIM1 and SIM3 and for SIM2 and SIM4, populations were grouped together in pairs in the table.

#### Real Data set

A real data set containing information from the performance test evaluations of young bulls belonging to the Rendena cattle breed was provided by the National Breeders Association of Rendena (ANARE). ANARE also provided herdbook information about the whole population traced back to the 1950s, whereas genomic data of bulls were, in part, provided by ANARE (PSRN DualBreeding, www.dualbreeding.it) and, in part, obtained under academic funding (SID Project, BIRD183281). Rendena is a small local population (6,384 heads for 249 breeding males and 6,135 breeding females belonging to 202 herds censed on 31.12.2020; fao/dad.is.org) bred for the dual-purpose attitude of milk and meat. Rendena is native to the Northeastern Alps in Italy but is now widespread also in the adjacent lowland territory on the right side of the Brenta River in the Veneto region (Po Valley; Guzzo et al., 2018).

The real phenotypes considered in this study were single individual records of average daily gain (ADG), *in vivo* estimates of carcass fleshiness (CF) and dressing percentage (DP) collected in the years 1985–2020. These traits have been extensively described in Guzzo et al. (2019) and Mancin et al. (2021b). The Illumina Bovine LD GGP v3, comprising 26,497 SNP markers (low-density panel: LD), and Bovine 150K Array GGP v3 Bead Chip, including 138,974 SNPs (Illumina Inc, San Diego, CA, United States; high-density panel: HD), were used for genotyping Rendena cattle.

The LD panel belonging to 1,416 individuals with 26,497 SNPs was imputed on the HD panel with 138,974 SNPs belonging to 554 bulls. The overlap between the two panels was about 60%. Information about data quality control and imputation is reported in greater detail by Mancin et al. (2022). In addition to the previous study, further quality control was performed by removing SNPs with high linkage disequilibrium (>80), using SNPrune (Calus and Vandenplas, 2018): this removed a total of 28,049 SNPs. An amount of 85,331 SNPs was finally retained for analysis. Overall, the study considered 1,691 young bulls with only phenotypic information, 1,739 animals with only genotypic information, and 687 animals with both phenotypic and genotypic information. The data structure of the real data set used for genomic prediction is reported in Table 2.

### Prediction Models

The breeding values for the single trait of the four simulated populations and the three performance test traits of the real Rendena data set were estimated using several BLUP models. First, we used four ‘classical’ BLUP models:

1) standard pedigree best linear unbiased prediction (PBLUP, described in *Pedigree Best Linear Unbiased Predictor*);
2) single-step genomic BLUP (ssGBLUP, described in *Single-Step Genomic Best Linear Unbiased Predictor*);
3) small shrinkage weighted single-step genomic BLUP (WssGBLUP1, described in *Weighted Single-Step Genomic Best Linear Unbiased Predictor*);
4) high shrinkage weighted single-step genomic BLUP (WssGBLUP2, described in *Weighted Single-Step Genomic Best Linear Unbiased Predictor*).

Then, we performed five ssGBLUPs with preselected SNPs (described in 2.2.4). SNP selection was achieved using the following algorithms:

5a) least absolute shrinkage and selection operator [LASSO, described in *Least Absolute Shrinkage and Selection Operator (LASSO)*];
5b) spike-and-slab LASSO (SSLASSO, described in *Spike-and-Slab LASSO*);
5c) recursive feature elimination using ridge regression [RfeRR, described in *Recursive Feature Elimination Using Ridge Regression (RfeRR)*];
5d) recursive feature elimination using support vector machine [RfeSVM, described in *Recursive Feature Elimination Using Support Vector Machine (RfeSVM)*];
5e) extreme gradient boosting (XGBoost, described in *Boosting Ensemble*).

#### Pedigree Best Linear Unbiased Predictor

PBLUP was the first method used to estimate predictors, and it is described by the following equation (Henderson, 1975):

$$\left[\begin{array}{cc} X^{\prime }X & X^{\prime }Z\\ Z^{\prime }X & Z^{\prime }Z+A^{-1}\frac{\sigma_{e}^{2}}{\sigma_{a}^{2}} \end{array}\right]\left[\begin{array}{c} \hat{\mu }\\ \hat{a} \end{array}\right]=\left[\begin{array}{c} X^{\prime }y\\ Z^{\prime }y \end{array}\right]$$

where **y** is the vector of phenotypic observations, **X** is the matrix of the incidence of fixed effects, and **b** is the vector of these effects. In the real data set, fixed effects are represented by the contemporary group (young bulls tested at the same period in the same pen; 142 levels) and parity group of dams in four classes (Guzzo et al., 2019). In the simulated data sets, **X** was substituted by a vector of **1**’s; thus, **b** stands for the mean of the models. Matrix **Z** represents the incidence matrix that relates the random genetic additive effect, included in vector a , to the phenotype. The random residual error was included in a vector **e** showing normal distribution $N\left(0,I\sigma_{e}^{2}\right)$ **,** where $\sigma_{e}^{2}$ is the residual variance. The vector of additive genetic effects is distributed as $N(0,A\sigma_{a}^{2}$ ), where $\sigma_{a}^{2}$ is the genetic variances and **A** is the identical by descent (IBD) relationship matrix constructed from pedigree data.

#### Single-Step Genomic Best Linear Unbiased Predictor

We used ssGBLUP as a benchmark to evaluate the impact of other models (see further, WssGBLUP and ssGBLUP with selected SNPs). The ssGBLUP method presents the same structure of equation as in 2.2.1, except for the (co)variance matrix of random genetic effects, which is substituted by **H**, as described by Aguilar et al. (2010):

$$H^{-1}=\;A^{-1}+\;\left[\begin{array}{cc} 0 & 0\\ 0 & G^{-1}-A_{22}^{-1} \end{array}\right]$$

where A and $A_{22}^{-1}$ are the reverse of the IBD matrix for all animals and for only genotyped animals, respectively, and **G** is the genomic matrix including the genomic relationships among animals.

The **G** matrix was built using the first methods proposed by VanRaden (2008b):

$$G_{0}=\frac{MM^{\prime }}{2\sum p_{i}\left(1-p_{i}\right)}$$

where *p* is the allele frequency of the *i*th locus and **M** is a matrix of SNP content centered by twice the current allele frequencies. Since the frequencies of the current genotyped population are used to center **G**, pedigree and genomic matrices have different bases, **G** was adjusted so the average diagonal and off-diagonal matched the averages of diagonal and off-diagonal in **A** ^**22**^, as described by Vitezica et al. (2011).

#### Weighted Single-Step Genomic Best Linear Unbiased Predictor

The WssGBLUP is the third method we used (two models, each with a different CT value, as explained below). This approach is equal to model 2.2.2, except for the matrix **G,** built following the second method of VanRaden (2008a), as shown below:

$$G_{0}=\frac{MDM^{\prime }}{2\sum p_{i}\left(1-p_{i}\right)}$$

where *p* is the allele frequency of the *i*th locus, **M** is a matrix of SNP content centered by twice the current allele frequencies, and **D** is the diagonal matrix in which SNP specific weights are contained. The iterative algorithm reported by Zhang et al. (2016) has been used as a weighting strategy. The SNP weights presented in **D** were obtained as a function of the estimated SNP effect ( $\hat{u}$ ). The weighting function used in this study was called non-linear A, as reported by Fragomeni et al. (2019). This method was preferred over other weighting strategies due to its stability among the iterations. The iterative algorithm applied followed the steps reported below:

1. The initial parameter was set as $t=1,\;D_{\left(t\right)}=I,G_{\left(t\right)}=\frac{MD_{\left(t\right)}M^{\prime }}{2\sum p_{i}\left(1-p_{i}\right)}$ , where **I** is an identity matrix;
2. GEBV ( $\hat{a})$ is obtained**,** where $\hat{a}$ is the vector of solutions of additive genomic breeding value using the ssGBLUP algorithm;

3. The SNP effect ( $\hat{u})\;$ is obtained, as in Gualdrón Duarte et al. (2014):

$$\hat{u}=\frac{1}{2\sum p\left(1-p\right)}DM^{\prime }{\left[MDM^{\prime }\right]}^{-1}\hat{a}.$$

4. $d_{i\left(t+1\right)}$ , as in Fragomeni et al. (2019), is transformed in ${\text{CT}}^{\frac{\left|{\hat{u}}_{i}\right|}{sd\left(\hat{u}\right)}-2}$ , where CT is a shrinkage factor determining how much the SNP effect distribution deviates from normality;
5. The weight of SNPs is standardized by maintaining a constant genetic variance among iterations:

$${\text{D}}_{\left(\text{t}+1\right)}=\frac{\text{t}\text{r}\left({\text{D}}_{\left(1\right)}\right)}{\text{t}\text{r}\left({\text{D}}_{\left(t+1\right)}\right)}\text{t}\text{r}\left({\text{D}}_{\left(t+1\right)}\right).$$

6. Matrix **G** is then recreated by including the new weights: $G_{\left(t+1\right)}=\frac{\text{M}{\text{D}}_{\left(t+1\right)}{\text{M}}^{\prime }}{2\sum p_{i}\left(1-p_{i}\right)}$ ;
7. Set  t=t+1 and go to point 2 for a new iteration.

We created two different WssGBLUP models with two different CT values: WssGBLUP1 had a CT value of 1.105, while WssGBLUP2 had a CT value of 1.250. This process was carried out to grant WssGBLUP1 the lowest possible shrinkage effect and WssGBLUP2 the highest possible shrinkage effect. For both models, the maximum number of iterations was set to five. For simplicity, we reported only two WssGBLUP predictions instead of the 10 analyzed in this study (combination of two CT values and five iterations). Thus, we retained two opposite WssGBLUP scenarios: WssGBLUP1, which presents the lowest SNPs shrinkage effect, and WssGBLUP2, which provides the highest shrinkage effect.

#### Single-Step Linear Unbiased Predictor With Only Informative SNPs

The last group of models (five models) consisted of ssGBLUP in which the **G** matrix of 2.2.2 was constructed using SNPs obtained after applying the different variable selection algorithms (described below, Section 2.4). The number of columns in **Z** is, thus, different for each trait and each data set.

#### Model Computations

**A** was built with the pedigree information tracking back up to three generations in all models. In addition, according to Cesarani et al. (2019), the variance components of each data set were estimated under PBLUP models by tracing back all animals in the pedigree. Variance components were estimated using the AIReml algorithm (Gilmour et al., 1995). All genetic and genomic prediction analyses were performed using the BLUPF90 family of programs (Aguilar et al., 2018). The consistency of all this information is reported in Table 2. Preliminary analysis, such as LD calculation, was conducted using preGSf90 (Aguilar et al., 2018, belonging to the BLUPF90 family of programs).

### Featured Selection Algorithms

The EBVs of the target trait were used to map the major SNP markers associated with the phenotype, using five different statistical approaches. The genome content was considered a covariance matrix, while EBVs of genotyped animals $\left(\hat{a}\right)$ (estimated using models in 2.2.2) were considered as the observed variable. The genome content was scaled in advance. Hyperparameter search and the choice of best models were performed by dividing the data set into a training group and a test group. In the real data set, young animals born after 2015 belonged to the test group, while older animals belonged to the training group. In the simulation, animals of 8th to 9th generations were part of the training group, while animals of the 10th generation belonged to the test group.

#### Least Absolute Shrinkage and Selection Operator

In the high-dimensional information literature, many penalized likelihood approaches have been proposed. Given the baseline $y_{i}=\;\beta_{0}+\overset{p}{\underset{j=1}{\sum }}x_{ij}\beta_{j}+e_{i}$ , a variant of the penalized likelihood approach can be described as follows:

$$\hat{\beta }=argmax-\frac{1}{2}\|\overset{N}{\underset{i=1}{\sum }}\{y_{i}-\;\left(\beta_{0}+\overset{p}{\underset{j=1}{\sum }}x_{ij}\beta_{j}\right){\|}_{2}^{2}+\;pen_{\lambda }\left(\beta \right)$$

where N is the number of animals for each trait, $\beta_{0}$ is model mean, $\beta_{j}$ is SNP contribution, p is the number of columns in x, N is the number of data, λ is the regularization parameter; and $pen_{\lambda }\left(\beta \right)$ is a penalty function. In LASSO (Tibshirani, 1996), the penalty is as follows:

$$pen_{\lambda }\left(\beta \right)=-\text{ λ}\overset{p}{\underset{j=1}{\sum }}\left|\beta_{j}\;\right|$$

A grid search was performed to find the optimal values obtained by testing values from 0 to 20 in increments of 0.1. These values were used to maximize the LASSO model performance, based on the highest coefficient of determination and the lowest mean squared error (MSE) in the training set. To carry out this calculation, we used the glmet R package (Friedman et al., 2010).

#### Spike-and-Slab LASSO

Spike-and-slab LASSO (SSLASSO) was proposed by Ročková and George (2018). It is based on the idea that every penalized likelihood has a Bayesian interpretation (Bai et al., 2021). For instance, the LASSO penalization is equivalent to a Laplace distribution regulated by hyperparameter λ , where the posterior mode of β is as follows:

$$p\left(\beta \text{|λ}\right)=\;\overset{p}{\underset{j=1}{\prod }}\frac{\text{λ}}{2}e^{-\text{λ}|\beta_{j}|}$$

The SSLASSO is the equivalent to a two-point mixture of Laplace distributions defined as follows:

$$p\left(\beta \text{|λ}\right)=\overset{p}{\underset{j=1}{\prod }}\left[\left(1-\gamma_{j}\right)\left(\frac{\text{λ}}{2}e^{-{\text{λ}}_{0}\left|\beta_{j}\right|}\right)+\gamma_{j}\left(\frac{\text{λ}}{2}e^{-{\text{λ}}_{1}\left|\beta_{j}\right|}\right)\right]\;$$

where $p\left(\gamma \text{|}\theta \right)=\overset{p}{\underset{j=1}{\prod }}\left[\theta^{\gamma_{j}}{\left(1-\theta \right)}^{1-\gamma_{j}}\right]$ and p(θ) ∼ Beta[a,b].

The Bayesian prior can be rearranged in a penalized likelihood context by taking this marginal logarithm as a prior (Bai et al., 2021); after some derivation, the following can be obtained:

$${\text{λ}}_{\theta }\left(\beta_{j}\right)=\;{\text{λ}}_{1}p_{\theta }\left(\beta_{j}\right)+\;{\text{λ}}_{0}\left[1-p_{\theta }\left(\beta_{j}\right)\right]$$

where

$$p_{\theta }\left(\beta_{j}\right)=\frac{1}{1+\frac{\left(1-\hat{\theta }\right)}{\hat{\theta }}\frac{{\text{λ}}_{0}}{{\text{λ}}_{1}}\exp\left[-\left|\beta_{j}\right|\left({\text{λ}}_{0}-{\text{λ}}_{1}\right)\right]}$$

SSLASSO was computed using the SSLASSO R package (Ročková and George, 2018), error variances were assumed to be unknown, and a self-adaptive penalty was set. In so doing, θ was assumed to be random and different shrinkage was applied to each $\beta_{j}$ .

#### Recursive Feature Elimination Using Ridge Regression

Similar to LASSO, ridge regression is based on a principle of penalized likelihood, with a penalty equal to $\text{λ}\overset{p}{\underset{j=1}{\sum }}\beta_{j}$ . Before proceeding with recursive feature elimination, the optimal values of λ were obtained as in LASSO selection. The glmet R package was used (Friedman et al., 2010).

After that, a recursive feature elimination using penalized ridge regression was performed as follows. In each iteration, the SNP effect $\beta_{j}$ was estimated based on training data. Then, 10% of the variable with lowest $\left|\beta_{j}\right|$ was removed from the subsequent iterations. The variable (SNP) present in the iteration with the lowest mean squared error (MSE) was considered for the prediction. MSE was calculated as ${\left(y_{test}-{\hat{y}}_{test}\right)}^{2},$ where $y_{test}$ is the EBV which belongs to the test database and $y_{test}$ is the predicted one.

#### Recursive Feature Elimination Using the Support Vector Machine

The SVM is a kernel-based supervised learning technique, often used for regression analysis. Depending on the kernel function considered, the SVM can map linear or nonlinear relationships between phenotypes and SNP markers. The best kernel function to map genotype to phenotype was determined in different training subsets: a five-fold split was used to determine which kernel function was a better fit for the data, either with linear, polynomial, or radial basis. We found that performing the SVM with a linear basis function outperformed the polynomial and radial basis function of about 12.5% in predictive ability.

The general model for the SVM (Evgeniou and Pontil, 2005; Hastie et al., 2009) can be described as follows:

$$y_{i}^{*}=b+h\left(m\right)*w+e$$

where h(m) represents the linear kernel basis function ( h(m)=m'm ) used to transform the original predictor variables (i.e., SNP marker information ( m )), b denotes model bias, and w represents the unknown weight vector. In the SVM model, the learn function h(m) was given by minimizing the loss function as follows: $C\overset{N}{\underset{i=1}{\sum }}L\left(y_{i}^{*}-{\hat{y}}_{i}^{*}\right)+\frac{1}{2}\|w{\|}^{2}.$ The C represents a regularization parameter, which controls the trade-off between predictor error and model complexity, and $w^{2}$ denotes the squared norm under a Hilbert space. The SVM model was fitted using an epsilon-support vector regression that ignores residual absolute value ( $\left|y_{i}^{*}-{\hat{y}}_{i}^{*}\right|$ ) smaller than some constant (ε) and penalizes larger residuals (Vapnik, 2000). The parameters C and ε were defined using the training data set as proposed by Cherkassky and Ma (2004): $C=\max\left(\left|\bar{y^{*}}+3\sigma_{y^{*}}\right|,\left|\bar{y^{*}}-3\sigma_{y^{*}}\right|\right)$ and $\varepsilon =3\sigma_{y^{*}}\left(\sqrt{\ln\left(n\right)/n}\right),$ where $\bar{y^{*}}$ and $\sigma_{y^{*}}$ are the mean and standard deviation of the target EBV for the traits on the training population, respectively, and *n* represents the number of animals in the training set. The SVM was performed using the e1071 R package (Meyer et al., 2020).

After that, recursive feature elimination using the SVM was performed using the same procedure described for RfeRR in the study by Sanz et al. (2018).

#### Boosting Ensemble

The boosting approach (XGBoost) is an ensemble technique that combines gradient descent error minimization with boosting, aiming to convert weak regression tree models into strong learners (Hastie et al., 2009; Natekin and Knoll, 2013). This ensemble process combines different predictor variables sequentially in the regression tree model, using regularization *via* selection and shrinkage of the predictors to control the residual from the previous model (Friedman, 2002). In addition, the XGBoost can use parallel computation to use more regularized models to prevent overfitting. The XGBoost approach can be described as follows:

$$y=\overset{W}{\underset{w=1}{\sum }}\beta_{w}h\left(x,\gamma_{w}\right)+e\;$$

where y is the vector of the target EBV; W is the number of iterations (expansion coefficients); $\beta_{w}$ is shrinkage factor, also known as “boost”; $h\left(x,{\text{γ}}_{w}\right)$ is base learner, a function of the multivariate argument x with a set of parameters ${\text{γ}}_{w}=\left\{{\text{γ}}_{1},\;{\text{γ}}_{2},\ldots ,{\text{γ}}_{w}\right\}$ ; and e is the vector of the residuals. Expansions of the coefficients ${\left\{\beta_{w}\right\}}_{1}^{W}$ and parameters ${\left\{{\text{γ}}_{w}\right\}}_{1}^{W}$ are used to map the predictor variables ( x ), that is, SNP markers to the target EBV ( y ) considering the joint distribution of all values (y,x) and minimizing the loss function $L\left\{y_{i},F\left(x\right)\right\}$ given as $\left[y,F_{w-1}\left(x_{i}\right)+h\left(y_{i};x_{i},p_{w}\right)\right],$ where $p_{w}$ is the predictor to minimize $\overset{n}{\underset{i=1}{\sum }}L\left[y,F_{m-1}\left(x_{i}\right)+h\left(y_{i};x_{i},p_{m}\right)\right].$ Our XGBoost follows the algorithm specified by Chen and Guestrin, 2016. In the XGBoost method, a regularization term is added in the loss function, representing the weight vectors learned in the loss function: this term penalizes the ponderation of large weights. This regularization term is defined as follows: $\overset{n}{\underset{i=1}{\sum }}L\left[y,F_{m-1}\left(x_{i}\right)+h\left(y_{i};x_{i},p_{m}\right)\right]+\underset{n}{\sum }\text{Ω}\left(f_{n}\right),$ where L is the error between the true value of the target trait and the predicted value and $\text{Ω}\left(f_{n}\right)$ is the regularization function used to prevent overfitting: $\text{Ω}\left(f_{n}\right)=\;\gamma T+0.5\lambda \omega^{2},$ where *T* is the number of leaves in the regression tree $f_{n}$ and ω represents the weight for the leaves in each tree (i.e., the predicted values stored at the leaf nodes). Including in the objective function makes the tree less complex, which minimizes the loss function and helps reduce overfitting; γT is a constant penalty for each additional tree leaf, and $\lambda \omega^{2}$ penalizes extreme weights. The γ and λ are the regularization terms L1 and L2, respectively (Mitchell and Frank, 2017). The random search for XGBoost was performed considering the four most important parameters able to increase prediction accuracy and minimize the prediction error. These hyperparameters were Ntree (total number of trees in the sequence used in the model), learning rate (determines the contribution of each tree to the final model and performs shrinkage to avoid variable overfitting), maximum tree depth (controls the depth of the individual trees to be considered in the model), and minimum samples per leaf (controls the complexity of each tree). The Ntree values ranged from 600 to 5,000 in intervals of 200; the learning rate was in the range of 0.05–1 in intervals of 0.05; the maximum tree depth was determined with a value ranging from 5 to 80 in intervals of 5; the minimum sample per leaf was set from 5 to 100 in interval*s* of 5 and considering lambda and alpha regularization values ranging from 0 to 1 in intervals of 0.05. The random grid search XGBoost was performed using the *h2o.grid* function of the h2o R package (https://cran.r-project.org/web/packages/h2o), considering as fixed parameter a maximum of 150 models with random combinations of the hyperparameters over 60 min.

### Effective Population Size Calculations

The effective population size (N_e_) has been computed from the individual increase in inbreeding ( ΔF) (Falconer and Mackay, 1996) to compare real and simulated data properly. Individual ΔF was calculated as follows:

$$\text{Δ}F=\frac{F_{n}-F_{n-1}}{1-F_{n-1}}$$

$$Ne=\frac{1}{2\text{Δ}F}$$

where $F_{n}$ is the inbreeding in the *nth* generation. Ne was calculated using the purge R package (https://cran.r-project.org/web/packages/purgeR).

### Validation

#### Validation in the Simulated Data set

Quality of prediction was measured as the correlation and MSE between the genomic breeding values estimated under different models and the true breeding values for animals belonging to the 10th generation, that is, the last generation of animals, including individuals without phenotypes but with genotype.

#### Validation in the Real Data set

In the real data set, two different cross-validation methods were applied. The first method we used to cross validate predictive ability was to calculate both the correlation and MSE between predicted and observed phenotypes. In this case, five-fold cross-validation with 10 iterations was performed. Since not all animals were genotyped in each iteration, 1/5 of non-genotyped and 1/5 of genotyped animals were masked. The current study considered predictive ability metrics only for genotyped animals; however, results about non-genotyped animals were also obtained (Supplementary Figure S1).

Linear regression (LR) (Legarra and Reverter, 2018) was used as the second cross-validation method. It compares the prediction performances of different models on groups of focal individuals born after a given date, in this case, the young bulls. LR is particularly suited to the specific needs of the Rendena population since predicting the future performance of young bulls without phenotype is one of the main objectives of the breeding plans for performance tests (Mancin et al., 2021a).

The LR method evaluates the goodness of a model by comparing its performance in a complete data set and a partial data set. The complete data set contains the whole amount of information or it is the data set used for prediction. A partial data set is referred to as the complete data set with some animals with the phenotype removed, usually young animals known as candidates to selection. According to Macedo et al. (2020), we built partial data sets by excluding phenotypes since a target recent birth year of young bulls (since 2012–2020; since 2014–2020; and since 2017–2020) to describe possible variations and random deviations of the estimator; consistencies are reported on Table 3. LR considered the following three parameters: bias, dispersion, and accuracy. Bias is the difference between the expected breeding values estimated under the complete and partial data sets. Dispersion was calculated as the regression coefficient considering the breeding values from the complete data set on the ones estimated from the partial data and accuracy as correlations between the two breeding values.

**TABLE 3:** Description of different validation sets used in cross-validation. The first and last years of birth of animals in the training data set and the number of animals (individuals) used in the validation cohort are reported.

**First**	**Last**	**Individuals**
2012	2020	178
2013	2020	154
2014	2020	130
2015	2020	109
2016	2020	106
2017	2020	72
2018	2020	45

## Results

### Genomic Structure

#### Genomic Structure in Simulated Data sets

**FIGURE 2:**
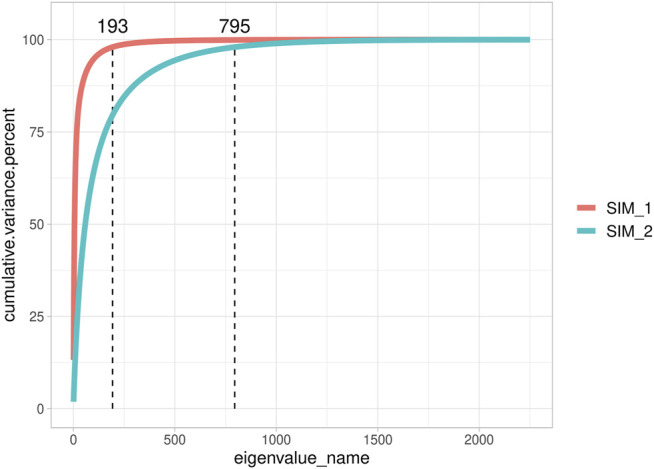
Cumulative explained variance of all eigenvalues of the genomic relationship matrix of two representative simulated populations.

Figure 2 highlights the different genomic assets of small Ne populations (SIM1 and SIM3; 10 sires per generation) and large Ne populations (SIM2 and SIM4; 200 sires per generation). Since the different number of QTLs assumed for the populations with the same Ne (that is, 10 vs. 1000 QTL) did not impact **G** matrix dimensionality, only SIM1 and SIM2 were plotted for simplicity. In SIM1, 193 eigenvalues were necessary to explain 98% of **G** matrix variance, while in SIM2, 795 eigenvalues were necessary to explain 98% of **G** matrix variance. When only ten sires per generation were used, it was possible to observe different subpopulations (Supplementary Figure S2); however, no population structure was found when plotting the first two eigenvalues. On the other hand, SIM2 appeared homogenous, and individuals seemed almost unrelated. In addition, when LD per chromosome was calculated, a greater value was observed in SIM1 (0.161 ± 0.076) than that in SIM2 (0.067 ± 0.054; data not shown). An Ne value of 81.18 ± 4 was determined for SIM1 and SIM3 and 1869 ± 546 for SIM2 and SIM4.

#### Genomic Structure in the Real Data set

**FIGURE 3:**
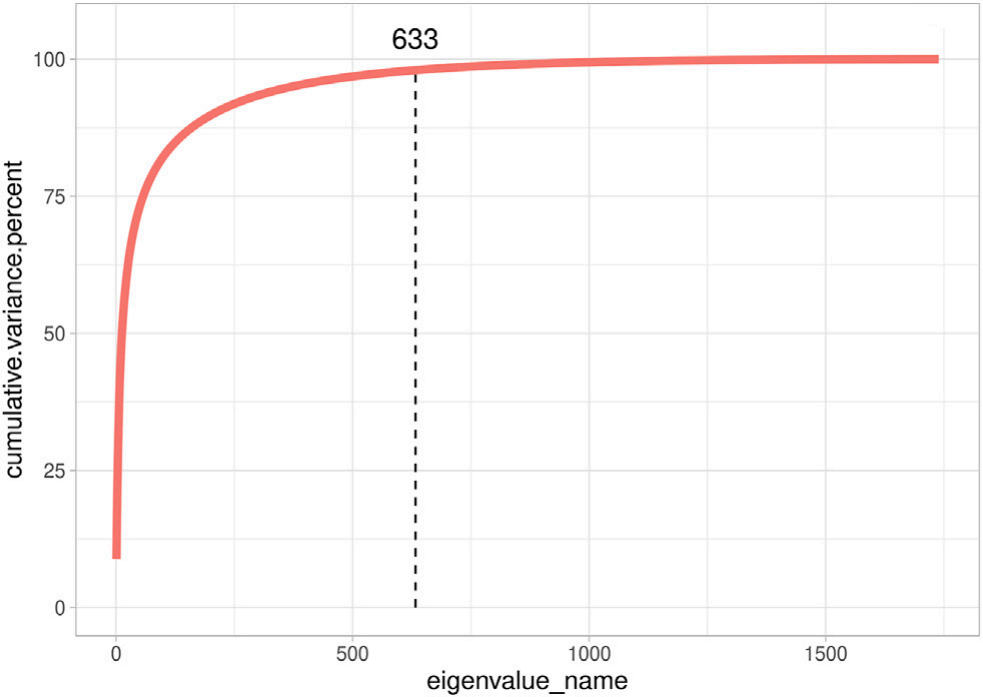
Cumulative explained variance of all eigenvalues of the genomic relationship matrix of Rendena populations.

We also investigated **G**’s dimensionality on the real data set of the Rendena cattle population (Figure 3). The real data set presented a situation closer to SIM1 and SIM3 than to SIM2 and SIM4. It showed, indeed, an average Ne value of 108.2 ± 0.74 calculated from pedigree data. It is possible to observe a few clusters in the genomic relationship matrix (Figure 3); however, they are not as straightforward as in SIM. We, therefore, can note that no population structure is present in Rendena breed (i.e., no subpopulations), which is in line with previous research (Mancin et al., 2022). Only 633 eigenvalues explained 98% of G variance; thus, the scenario was closer to SIM2 than SIM1. In addition, we observed an average LD of 0.187 ± 0.107 per chromosome (Mancin et al., 2022).

### SNPs Retained by Variable Selection Models

#### SNPs Retained in Simulated Data sets

**FIGURE 4:**
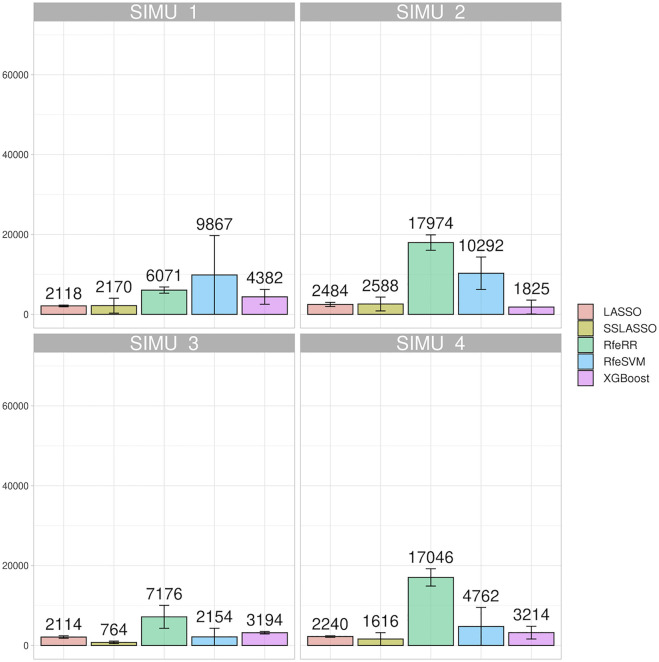
Bar plot representing the number of SNPs retained by each algorithm on the four simulated population; error bars represent standard deviation.

Figure 4 reports the impact of the different algorithms in terms of the number of informative markers retained. Specifically, we were interested in identifying the impact that different **G** matrix dimensionality and number of QTLs had on the number of SNPs considered informative. In all simulations, LASSO and SSLASSO retained the lowest number of SNPs (roughly 2,000 SNPs averaged across simulations), and they presented lower intra- and between-scenario variability. On the contrary, RfeSVM and RfeRR algorithms retained higher numbers of SNPs, on average 12,000 for RfeRR and 7,000 for RfeSVM. RfeSVM also presented an extreme variability across scenarios (Figure 4). XGBoost retained an intermediate number of SNPs, with an average of 3,000 SNPs retained across simulations. As shown in Figure 4, different numbers of QTLs did not affect the number of SNPs retained by each algorithm. In fact, no difference was observed between SIM1 vs. SIM3 and SIM2 vs. SIM4; only LASSO and SSLASSO algorithms seem to be slightly affected by the number of QTLs. Interestingly, the dimensionality of the **G** matrix seems to be more influential as scenarios with higher Ne presented a higher number of SNPs (SIM1 and SIM2). The XGBoost is the only algorithm where this trend was not seen. Crucially, we observed that the negative gap in model accuracy present in simulations with lower QTL (SIM3 and SIM4) fades when variable selection models are introduced.

#### SNPs Retained in the Real Data set

**FIGURE 5:**
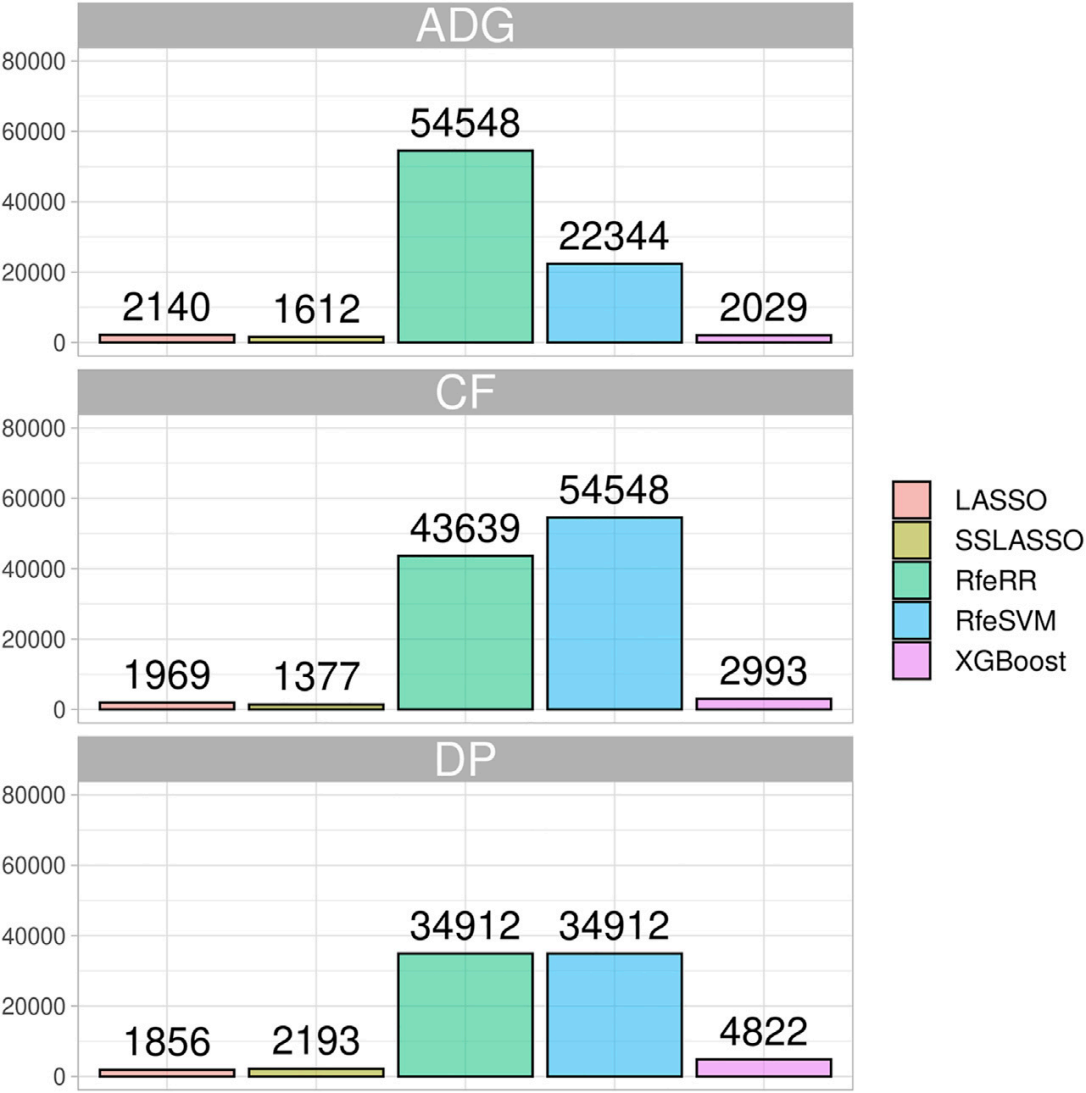
Bar plot representing the number of SNPs retained by each algorithm on the three phenotypes of the Rendena population.

We showed the impact of variable selection methods regarding the number of informative markers retained in the Rendena population in Figure 5. Although the number of initial SNPs was similar to that of the simulated populations, in general, the algorithms retained a higher number of SNPs in the real data set. Similar to what was reported in the simulated data, LASSO and SSLASSO were the most restrictive algorithms of SNP selection, with an average of 2,000 SNPs retained across the simulations. The XGBoost was the second most restrictive algorithm in terms of SNPs retained by the models, about 3,000 on average. RfeSVM and RfeRR algorithms retained almost half of the SNPs presented in the panels (40,000 SNPs). No clear patterns were identified across different phenotypes: some algorithms found a greater number of SNPs in certain traits and some in others. For example, the lowest number of informative markers retained by RFE algorithms was identified on the DP trait, but the opposite situation occurred for XGBoost, where the number of informative SNPs retained for DP was almost twice the number of informative SNPs retained for other traits.

### Breeding Value Prediction

We compared the prediction accuracy of four ‘classical’ models for BLUP and ssGBLUP with five different SNP preselection strategies. The models are detailed in *Materials and Methods* and summarized as follows: 1) PBLUP; 2) single ssGBLUP; 3) WssGBLUP1; 4) WssGBLUP2; 5a) ssGBLUP with SNPs preselected via LASSO; 5b) ssGBLUP with SNPs preselected via SSLASSO; 5c) ssGBLUP with SNPs preselected via RfeRR; 5d) ssGBLUP with SNPs preselected via RfeSVM; and 5e) ssGBLUP with SNPs preselected *via* XGBoost. Table 4 provides a qualitative summary of the results, described in the following paragraphs.

**TABLE 4:** Summary of results obtained using the nine models considered in the study and the cross-validations applied.

**Method name**	**Accuracy across simulations (Correlation/MSE)**	**Accuracy in real data set (Correlation/MSE)**	**Bias/Slope in LR cross-validation in real data set**
PBLUP	Poor	Poor	Good
ssGBLUP	Medium	Medium	Best
WssGBLUP1	Medium	Medium	Good
WssGBLUP2	Medium	Good	Poor
LASSO-selected ssGBLUP	Best	Best	Poor
SSLASSO-selected ssGBLUP	Best	Best	Poor
RfeRR-selected ssGBLUP	Good	Good	Poor
RfeSVM-selected ssGBLUP	Good	Good	Poor

#### Breeding Value Prediction in Simulated Data sets

**FIGURE 6:**
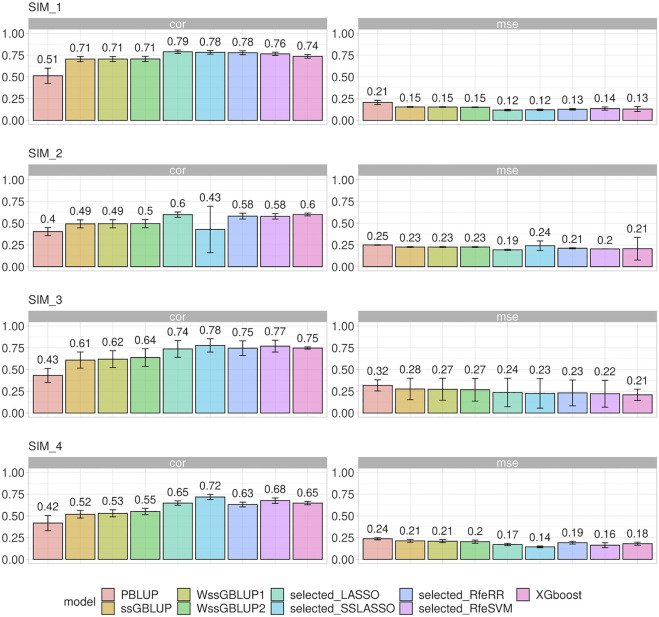
Bar plot representing correlation (corr) and mean squared error (MSE) between predicted and true breeding values on the four different simulated populations. Error bars represent standard deviations.

Different prediction model accuracies are reported in Figure 6, with correlation and MSE as comparison metrics. MSE values were comparable to those obtained for correlations. Standard BLUP models achieved the lowest accuracy. A substantial increase in accuracy was observed in ssGBLUP models, that is, when genomic data were integrated: this increase of accuracy was more relevant for populations with small Ne (SIM1 and SIM3).

A slightly greater accuracy than that in ssGBLUP was observed when a heterogenous distribution of SNPs was considered within the matrix **G** (WssGBLUP). The gap in accuracy was greater in the populations with few QTLs (SIM3 and SIM4), especially for WssGBLUP2. On the other hand, the increase in accuracy for SIM1 and SIM2 under WssGBLUP was almost close to zero. A substantial variation in accuracy values was observed when ssGBLUP was performed with **G** matrixes constructed with selected SNPs; however, the accuracy of the prediction performance of each variable selection model changed according to the simulation structure. Generally, SSLASSO presented the highest increase in accuracy among the genetic models in all simulations, except for SIM2, where we observed a dramatic drop in accuracy. On the other hand, LASSO achieved greater accuracy on both SIM1 and SIM2. Other algorithms presented an intermediate increase in accuracy among the genetic models in all simulations, namely, RfeRR, RfeSVM, and XGBoost, with different rankings in different scenarios.

#### Breeding Value Prediction in Real Data set

**FIGURE 7:**
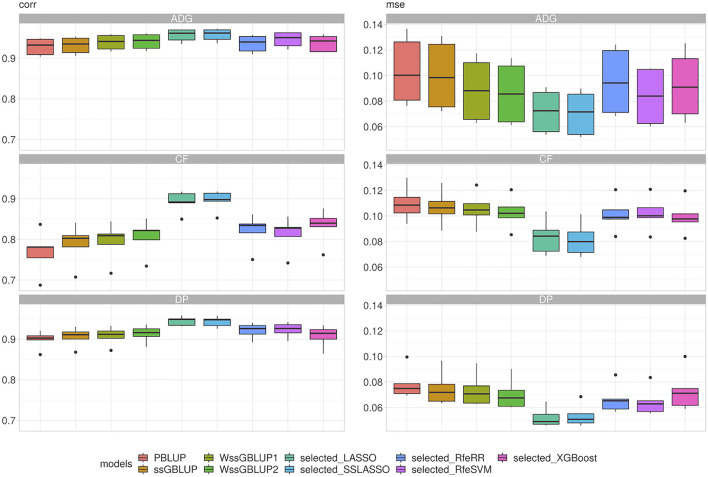
Box plot representing correlation (corr) and mean squared error (MSE) between predicted and true breeding values of phenotypes recorded in Rendena performance testing stations. Target phenotypes are ADG: average daily gain; CF: *in vivo* carcass fleshiness; DP: *in vivo* dressing percentage.

With our real data sets, we were first interested in evaluating the performance of these models in terms of prediction; then, we wanted to evaluate the feasibility of introducing them in a real breeding plan scenario. This point was achieved using LR cross-validation methods (Legarra and Reverter, 2018). Figure 7 presents the results of repeated five-fold cross-validation. The integrations of genomic data led again to a substantial increase in accuracy: the PBLUP presented the overall lowest correlation (r) values (r from 0.36 to 0.53). The ssGBLUP presented the lowest correlation values among genomic models (r from 0.46 to 0.62), while a slight increment was observed for WssGBLUP1 (from 0.55 to 0.67) and for WssGBLUP2 (from 0.67 to 0.75). As with simulated data, variable selection models improved model accuracy substantially. Again, the highest correlations were found for LASSO and SSLASSO, with values of r ranging from 0.83 to 0.92, while other algorithms presented intermediate values (r around 0.70). This pattern was observed across all traits. MSE reflected the results obtained with correlations.

**FIGURE 8:**
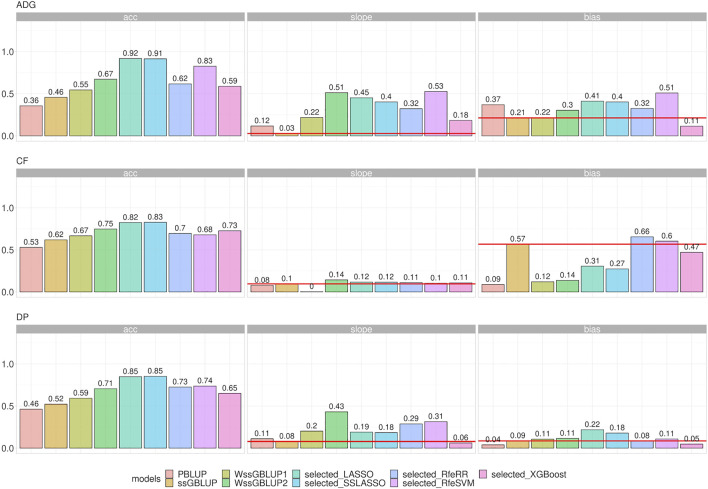
Bar plots representing accuracy, dispersion, and bias of the Rendena data set estimated using LR cross-validation in the validation cohort of 2015–2020. Dispersion was defined as 1, the absolute value of dispersion, while bias as absolute values of bias divided by its genetic variance to improve assessment of model rankings. Horizontal lines represent the values of ssGBLUP to permit easier comparison among models.

**FIGURE 9:**
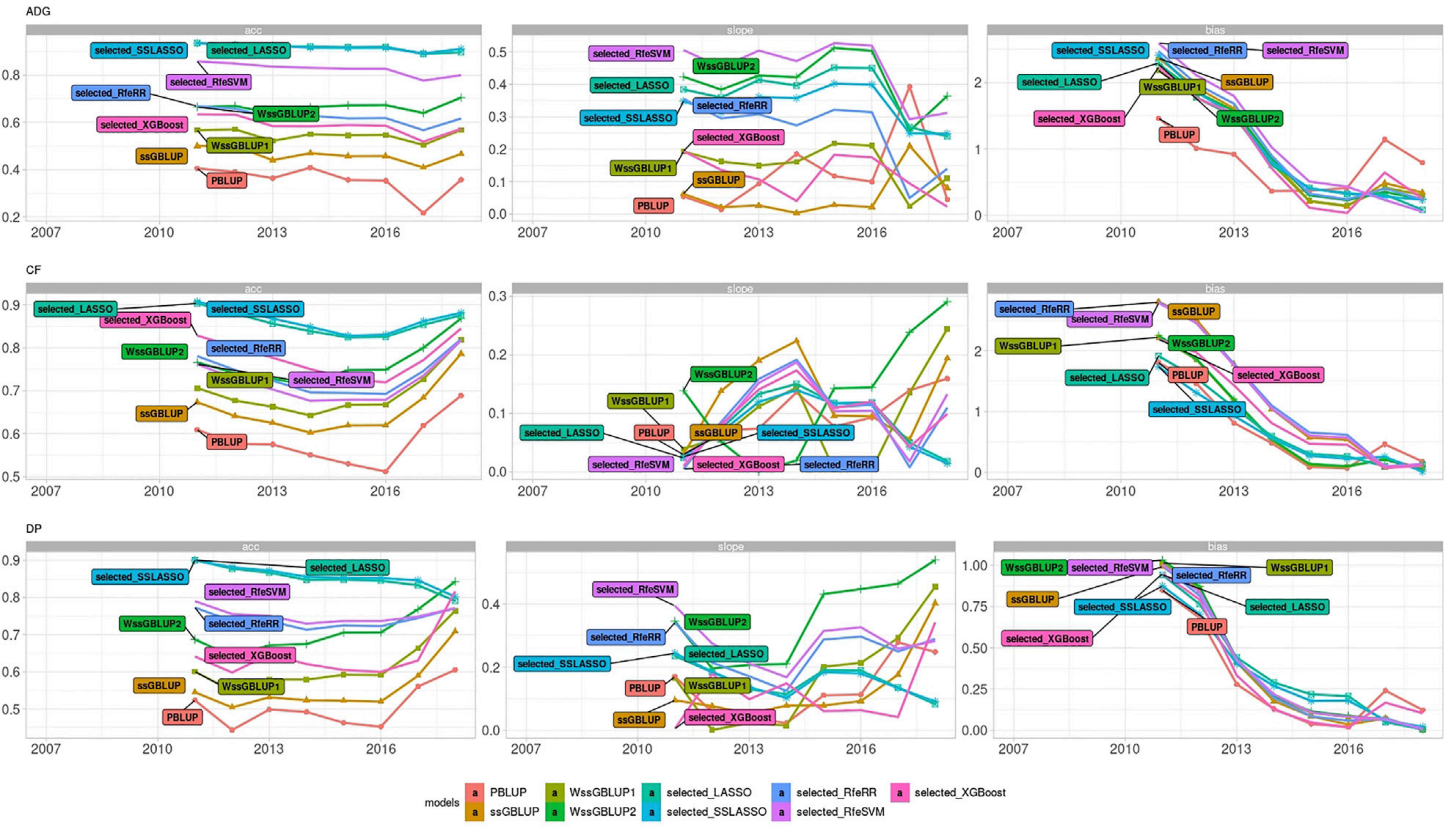
Line plot representing accuracy, dispersion, and bias of Rendena data set estimated using LR cross-validation in the validation cohort of 2021. Dispersion was represented as 1, absolute value of dispersion, while bias as absolute values of bias divided by its genetic variance.

LR methods evaluated dispersion and bias in addition to accuracy. Figure 8 represents the different results obtained using LR cross-validation methods in various validation sets from 2015–2020. This set of years was chosen as representative of all seven validation cohorts. Figure 9 reports the summary statistics of all seven validation cohorts.

Accuracy trends of the real data set measured with the LR method were similar to the accuracies obtained with five-fold cross-validation. However, looking at the other statistics (slope and bias), we can observe that LASSO, SSLASSO RfeRR, and RfeSVM cannot be considered suitable variable selection approaches in real breeding plans due to their higher bias and dispersion values, especially if compared with ssGBLUP. Conversely, XGBoost was the only model with similar or even lower bias and dispersion values than ssGBLUP but with greater accuracy. As seen in Figure 9, we demonstrate that these trends are consistent over different validation cohorts.

## Discussion

The present study had two objectives: testing if reducing the number of SNPs used to construct **G** could lead to an increase in the accuracy of (ss)GBLUP and whether this method could be introduced in genomic evaluations of a real population with a small size, such as the Rendena breed.

In our study, using both simulated and real data sets, we demonstrated that the accuracy of (ss)GBLUP increases when it is performed when the number of SNPs to construct **G** was reduced. This finding agrees with that of the extensive literature supporting the increased accuracy of Bayesian variable selection models in many different species (Lourenco et al., 2014; Mehrban et al., 2021; Yoshida et al., 2018; Zhu et al., 2021). For example, Akbarzadeh et al. (2021) integrated only a subset of chosen SNPs into the GBLUP framework based on a classical GWAS analysis (i.e., 1, 5, 10, and 50% of significant SNPs). A slightly greater accuracy than that in the canonical GBLUP was observed when **G** was constructed using only the best 10 and 50% SNPs; contrariwise, models using the 1 and 5% of the SNP prediction underperformed. Furthermore, Akbarzadeh et al. (2021) reported a dramatic decline in performance when the same percentage of SNPs was randomly chosen. We tried preliminary tests of a similar approach—construction of the **G** matrix using the top 500, 1,000, and 50,000 SNPs ranked by their absolute SNP effect values calculated through back solutions—in Rendena breed; however, we immediately discarded this approach because of the extreme bias and inflated breeding value predictions (these findings are reported by Mancin et al., 2022 in press). In addition, choosing so few and unrepresentative SNPs reduced a lot the compatibility between **A** and **G** matrices, and thus ssGBLUP properties were affected (Misztal et al., 2013).

Li et al. (2018) and then Piles et al. (2021) showed how using different methods to select the most informative SNPs could significantly improve the performance of the variable selection models. Li et al. (2018) constructed the **G** matrix using the best 400, 1,000, and 3,000 SNPs, ranking SNPs effects by three different machine learning models. As in the previous case, an increase in accuracy was obtained only with a certain number of selected SNPs (1,000 SNPs), while a lower accuracy than that in the canonical GBLUP was observed with a lower number of SNPs. In addition, Piles et al. (2021) and Azodi et al. (2019) showed that by combining different variable selection algorithms with various parametric and nonparametric prediction models (i.e., ensemble predictions), it is possible to obtain a consistent increase in accuracy compared to models without variable selection. However, our study has not explored these scenarios since prediction methods other than ssGBLUP or ssSNP-BLUP (Fernando et al., 2017) do not seem to bring any concrete improvement for livestock traits (Abdollahi-Arpanahi et al., 2020). Furthermore, ssGBLUP and ssSNP-BLUP are the only methods that allow combining straightforwardly non-genotyped animals with genotyped ones—a crucial feature for a real-life routine selection plan and something that the other algorithms cannot do.

Our result that reducing the number of parameters positively impacts accuracy is also supported by Frouin et al. (2020). In that study, it was demonstrated that the error of the prediction tends to linearly increase when n > p until the “irreducible” error $\left(1\;-\;h^{2}\right)$ occurring when n ≫  p. In addition, Pocrnic et al. (2019), demonstrated that the accuracy of (ss)GBLUP is connected by the distribution of eigenvalues of **G**; thus, “n” becomes the number of independent chromosome segments (Me) captured by SNPs (Pocrnic et al., 2019). In highly related populations (small Ne), higher accuracy values can be achieved than in populations with larger Ne because fewer eigenvalues and thus a small “n” are necessary to explain G. As a matter of fact, in large Ne populations, more data are needed to increase accuracy. This issue is also intuitive since prediction error accuracy (Henderson, 1975) is directly proportional to the coefficient $C^{aa}$ (defined below); thus, in highly related populations, $C^{aa}$ tends to have lower values. $C^{aa}$ is the inversion of the coefficient matrix of the mixed model equation where *aa* is the block referring to the genetic effect of animals. What was reported by Pocrnic et al. (2019) could explain the lower performance identified by Akbarzadeh et al. (2021) when subsets of 1 and 5% of SNPs were considered (Akbarzadeh et al., 2021). Indeed, discarding too many SNPs from the construction of **G** may omit the inclusion of important eigenvalues. From another perspective, Fragomeni et al. (2017) demonstrated the positive impact of removing non-informative SNPs on GBLUP. The authors showed in a simulated data set that better accuracy was found when the **G** was built by eliminating all SNPs outside the window where the QTL was situated or using only QTL information. However, a practical limit to this method is that knowing all the QTLs within a genome is nearly impossible, especially when the population is small (Mancin et al., 2021a).

Our simulated results support the abovementioned theory, as simulations with lower Ne presented higher accuracy of ssGBLUP (SIM1, SIM3). Furthermore, differences between scenarios emerge when comparing simulations differing for their number of QTLs. ssGBLUP showed lower performance in SIM3 and SIM4 (QTL10) than in SIM1 and SIM2 (QTL1000); however, this discrepancy in accuracy decreases by applying variable selection. This result agrees with that by Daetwyler et al. (2010), which demonstrated that SNP selection *via* Bayes B presents substantial advantages when the number of QTLs is small compared to the number of independent chromosome segments.

As mentioned above, Bayesian SNP regression, or (ss)GBLUP using a weighted realized relationship matrix (Tiezzi and Maltecca, 2015; Zhang et al., 2016), always provides higher prediction accuracy than models assuming homogenous variance among SNPs (GBLUP or SNP-BLUP). However this increase in accuracy is often connected with increases in bias, especially when time cross-validation is used (Mehrban et al., 2021), instead of five-fold or leave-one-out cross-validation (Zhu et al., 2021). However, when the goal is to achieve the “best predictor”, namely, a value closer as possible to real one, models assuming heterogenous variances and models with variable selection can be identified as the best models. They have, indeed, the highest MSE, intended as bias-variance trade-off (Gianola, 2013). In this regard, LASSO and SSLASSO, thus, appeared as “best models” for both simulated and real data. We showed that (SS)LASSO regression performs automatic feature selection, especially in the presence of linearly correlated features, such as SIM1 and SIM3, since their simultaneous presence will increase the value of the cost function. Thus, LASSO regression will try to shrink the coefficient of the less important SNPs to 0 to select the best features.

However, in real-life breeding scenarios, time cross-validation must be considered (Liu, 2010; Legarra and Reverter, 2017) as this procedure simulates the natural accumulation of information across time. Only a few studies evaluated the impact of heterogenous or variable selection models using time cross-validation with small samples of individuals. Cesarani et al. (2021) and Mancin et al. (2021b) found higher bias and overdispersion values in WssGBLUP than in ssGBLUP.

The same pattern emerged when we performed LR cross-validation (Mancin et al., 2021b; Cesarani et al., 2021), namely, that higher shrinkages or selected SNPs have high accuracy but carry higher bias and dispersion values. Specifically, (SS)LASSO models showed the best accuracy in all three traits when measured with LR. Conversely, other feature selection models and WssGBLUP presented lower accuracy. Among the variable selection models, we found slightly lower values of accuracy in the XGBoost; however, we suggest that XGBoost could be regarded as the best variable selection model among those tested as it is the only model that presented higher accuracy than ssGBLUP, at a net of better bias and dispersion.

Several questions persist about the use of these models in routine evaluation. One of these issues concerns the implementation of preselected SNPs in multitrait models. However, this is a recurring problem not only when the **G** matrix is built with preselected SNPs but also more in general whenever models take into account the specific genomic architecture of traits, as WssGBLUP does. A possible solution to bypass this issue might be using multiple **G** matrix prediction models, one for each trait: yet, this is not computationally straightforward. A preliminary selection of SNPs by multiobjective optimization framework algorithms, as in Garcia (2019), could be a more concrete approach for future studies.

Another possible concern about the large-scale use of variable selection ssGBLUP is the fluctuations of SNPs across generations. Similarly to the issue with multitrait models, this regards all genomic selections (Hidalgo et al., 2020); however, it is true that with respect to other methods, such as Bayesian SNP regression, generation-by-generation recalibration of SNP preselection algorithms can be highly computationally demanding, especially when algorithms such as XGBoost are chosen. Finally, SNP preselection could be influenced by variability in SNP frequency across animals or more in general in the presence of population structure, as with subpopulations. Nonetheless, in our study, the PCA plots referring to SIM1 (Supplementary Materials S2), where some clusters are present, show that variable selection models overcome this issue quite effectively. It would be interesting to choose one or more variable selection models in future studies and evaluate their impact on more stratified populations.

Besides increasing the EBV accuracies, developing an optimal strategy for SNP variable selection in high-density panels will be particularly useful in local breeds. It would in fact allow the use of informative but lower density and cheaper panels, accounting for the best SNPs suitable for the target trait and population. Furthermore, given that small breeds cannot attract the same level of technological investment as their cosmopolitan counterparts (e.g., Holstein), decreasing the costs of genomic selection could be critical to help guarantee their selection, and thus their survival.

Aside from the economic factors, the importance of developing *ad hoc* selection methods for small-population cattle, especially for local breeds, is of primary importance for their conservation. Maintaining genetic progress for the productive characters and at the same time keeping intact the genetic variability and the distinct characteristics of the breeds can be guaranteed through breeding plans implementing careful selection (Biscarini et al., 2015). These plans are needed to preserve genetic variability within livestock local populations, a goal which, in the medium term, is critical for the animal husbandry industry to ensure the conservation of native breeds, their productive and reproductive efficiency, health, survival, and overall resilience to future changing environmental pressures (Mastrangelo et al., 2014).

## Conclusion

Genomic information, especially the single-step GBLUP technique, has brought great improvements to selection and breeding decisions in livestock. However, these methods still present methodological issues when applied to populations with a small size, such as local and endemic cattle breeds. Our rigorous testing of different algorithms for variable selection of informative SNPs has highlighted that prediction accuracy of variable selection ssGBLUP (especially that of XGBoost) was greater than that of other ssGBLUP methods, without the inflated bias and dispersion that accompany the weighted ssGBLUP. Our use of machine learning models could thus represent a solution to the issue of genomic selection in small populations. Local cattle breeds are an often untapped resource of genetic diversity and have great potential to adapt to varying environmental conditions. The methods presented here might, thus, be used in their conservation, study, and increase their economic competitiveness.

## Data Availability

The datasets presented in this study can be found in online repositories. The names of the repository/repositories and accession number(s) can be found below https://datadryad.org/stash/share/O6ld-ZCZLhAUtXmVpOlXkhkffVagc1_Stfnqktk907w.
