# Supplementary material for: Improvement of Genomic Predictions in Small Breeds by Construction of Genomic Relationship Matrix Through Variable Selection
Source: Front Genet. 2022 May 18;13:814264. doi: 10.3389/fgene.2022.814264 (PMC9158133; doi:10.3389/fgene.2022.814264)

Supplementary Material

# Figure S1: Bar plots representing accuracy, dispersion and bias of Rendena Dataset estimated using LR cross validation, in the validation cohort of 2015-2020. Dispersion was represented as 1-absolute value of dispersion while bias as absolute value of bias, to improve assessments of models rankings. Only non-genotyped animals were considered in the validation.


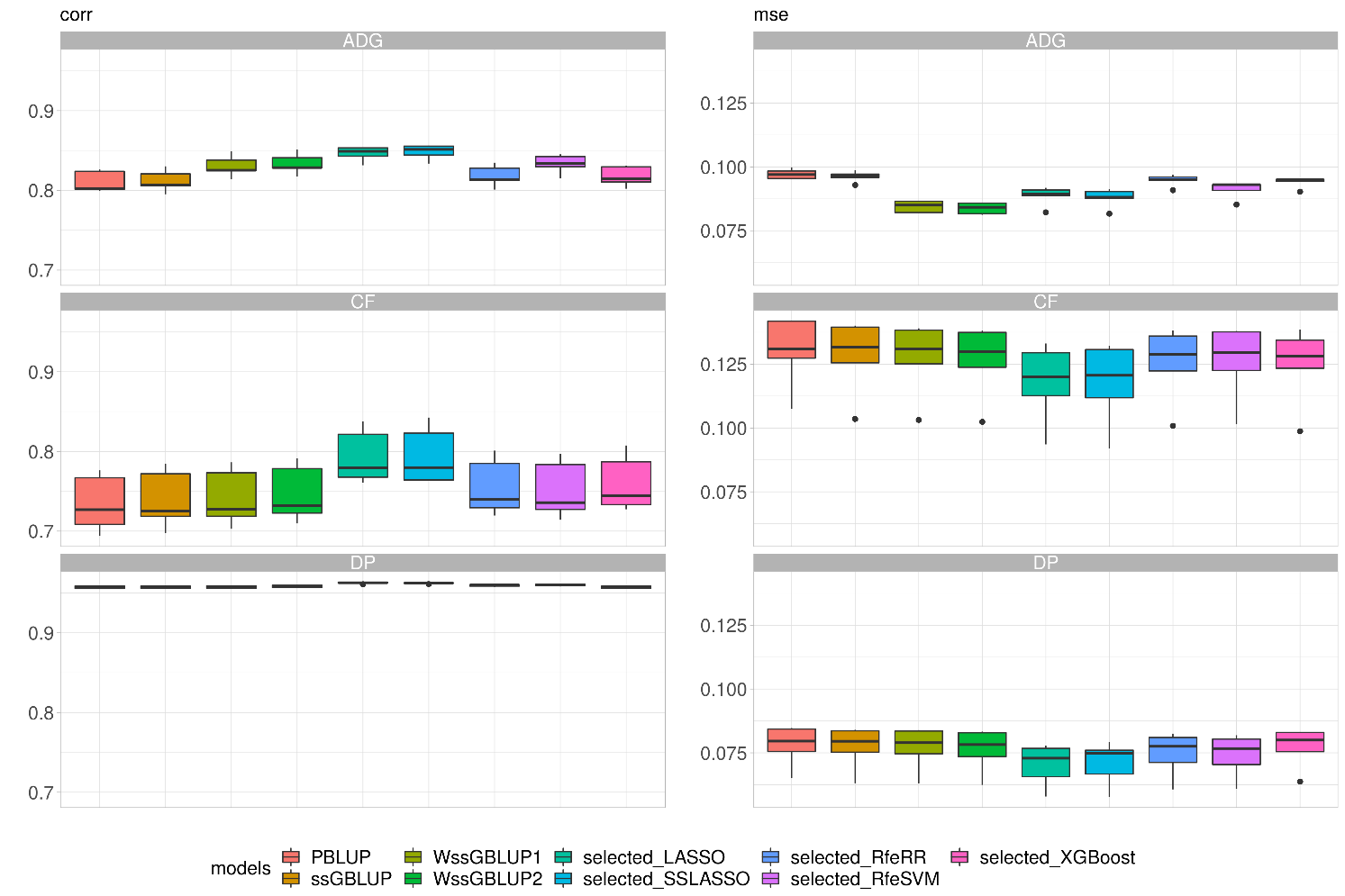


# Figure S2: PCA plots of the two simulated populations, SIM1 (to the left) and SIM2 (to the right).


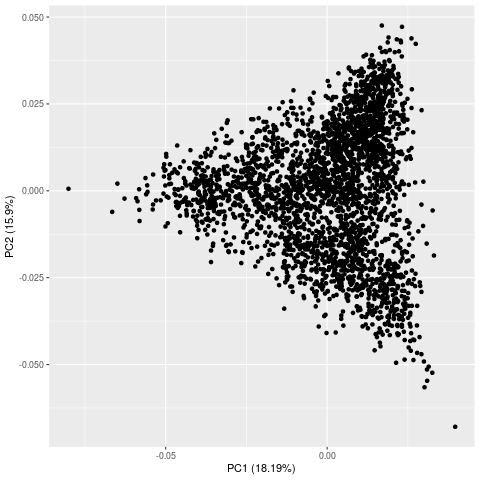

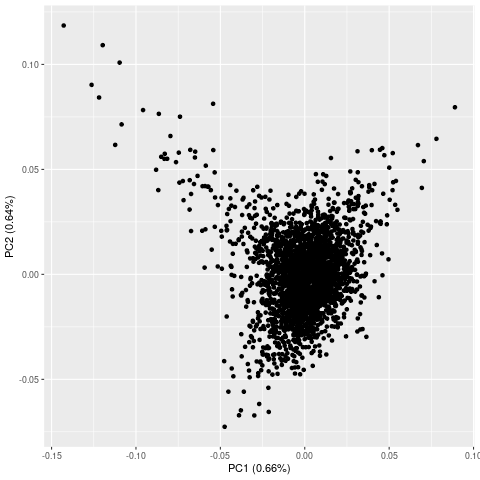

Supplement: Supplementary file 1 [file DataSheet1.docx]
